# Intravesical mitomycin C efficacy in acidic and alkaline urinary pH: impact on recurrence-free survival rate after TURBT

**DOI:** 10.1097/MS9.0000000000001350

**Published:** 2023-10-02

**Authors:** Muhammad Tayyab Naeem, Ahmed Hassan Usman, Sarmad Ali, Hassan Raza, Ali Nawaz Shah, Mohammed Mahmmoud Fadelallah Eljack

**Affiliations:** a Shaikh Zayed Hospital, Lahore; b Pak Oilfields Limited Hospital, Khaur, Attock; c Al-Shifa Hospital, Hospital Road, Mandi Bahuddin; d Gujranwala Teaching Hospital, Gujranwala, Pakistan; e University Hospitals of Birmingham, Birmingham, UK; f Faculty of Medicine and Health Sciences, University of Bakht Alruda, Ad Duwaym, Sudan

**Keywords:** carcinoma, medicine, mitomycin C, tumor recurrence, TURBT

## Abstract

**Background:**

Urinary bladder tumor recurrence following transurethral resection of bladder tumor (TURBT) is a common issue. This study aims to determine how urine alkalinization affects bladder tumor recurrence after surgery.

**Materials and methods:**

Sixty patients receiving mitomycin C (MMC) therapy after TURBT were divided into two groups based on mean pH values. Twenty-six patients were in group A, whose urine pH was below 5.5. However, there were 34 patients in group B, and their urine pH was higher than 5.5. Both groups of patients were given intravesical MMC once weekly for 6 weeks following TURBT. A cystoscopy was performed as a follow-up at 3, 6, and 12 months. Urine pH and the recurrence-free survival rate were compared using Kaplan–Meier survival analysis and the COX proportional hazard model.

**Results:**

The mean time to tumor recurrence in group A (intravesical MMC in acidic urine) and group B (intravesical MMC in alkaline urine) was 12.48 versus 16.84 months, respectively. Alkaline urine pH was identified as an independent predictor of preventing the recurrence of superficial bladder tumors by univariate COX regression analysis. Age, sex, and mean tumor size did not affect the likelihood of tumor recurrence. However, smoking had an association with increased tumor recurrence.

**Conclusion:**

Tumor recurrence post-TURBT is delayed in patients with alkaline urine pH. Smoking is an independent risk factor for bladder tumors.

## Introduction

Highlights

- Urinary bladder tumor recurrence following transurethral resection of bladder tumor is a common issue.
- Unlike smoking, age, sex, and mean tumor size did not affect the likelihood of tumor recurrence.
- Patients receiving intravesical mitomycin C with alkaline urine have improved recurrence-free survival rates compared to patients with an acidic urinary pH.

According to the most recent GLOBOCAN data, 3% of cancer diagnoses worldwide are for bladder cancer (BCa), which is more common in industrialized countries. BCa is the sixth most common malignancy in the USA^1^. BCa impacts men far more frequently than women worldwide, with incidence rates of 9.6/100 000 in men and 2.4/100 000 in women. BCa is the sixth most common and ninth most fatal malignancy in men^2^. Bladder tumor arise from cells lining the inner wall of the bladder. A cystoscopic examination is the most effective method for diagnosing BCa. Blue-light cystoscopy and narrow-band imaging are used for BCa staging^3^.

Bladder carcinoma is broadly classified as nonmuscle-invasive bladder carcinoma (NMIBC) and muscle-invasive bladder carcinoma (MIBC). The bladder tumor is staged as per TNM criteria. The pathological (pTNM) stage is supposed to provide details concerning the tumor’s dimensions, particularly local invasion, surgical margins, the extent of the lymphadenectomy, the location and number of metastatic nodes, and the tumor’s extracapsular spread^4^. NMIBC is one of the most prevalent types of BCa. Total of 75–85% of newly reported BCa cases are nonmuscle-invasive^5^. NMIBC is managed with transurethral resection of bladder tumor (TURBT) and adjuvant chemotherapy. NMIBC has a high recurrence rate of 25–40% in the first year following TURBT^6^. Adjuvant chemotherapy mainly consists of intravesical mitomycin C (MMC) to improve recurrence-free time^7–9^. Gemcitabine has a variety of pharmacologic characteristics that make it suitable for use as an intravesical treatment for NMIBC. First, *in vitro* developed BCa cells have shown that gemcitabine is active in destroying them. Second, the absorption into malignant urothelial cells is sufficient for cytotoxicity *in vivo* due to the low molecular weight and high lipid solubility. Bacillus Calmette–Guérin intravenous instillation is used to treat superficial BCa in order to lower the recurrence rate of bladder tumors and lower the risk of progression. In comparison to transurethral resection alone, Bacillus Calmette–Guérin prophylaxis after complete transurethral resection or fulguration of superficial disease considerably lowers recurrence and lengthens the disease-free period.

The transitional epithelium of the bladder wall is relatively impermeable to MMC. Various parameters like postvoid volume, urine production, and urine pH influence the efficacy of MMC. Sodium bicarbonate increases urinary pH, which can increase intravesical MMC stability^10–12^. Acidic urine is known to cause the recurrence of upper tract urothelial carcinoma and bladder tumor^13^. This study aimed to analyze the association between urinary alkalinization and preventing bladder tumor recurrence following TURBT.

## Materials and methods

Between 2021 and 2022, a retrospective study was performed on patients with superficial bladder tumors who underwent a 6-week course of intravesical MMC after TURBT. Approval was taken from the Institutional Review Board of SZH, Lahore (SZMC/TERC/298/2022). Only those cases were taken into consideration and administered MMC therapy whose excised tissue margins were both macroscopically and microscopically tumor-free. Patients older than 18 years and nonmuscle-invasive disease on histopathology were included in the current study, while those with prior diagnosis or treatment of bladder carcinoma, prior history of chemotherapy, history or current diagnosis of any malignancy, computed tomography scan findings consistent with muscle-invasive disease, patients who changed to other treatment plans, or ambiguity in the pH value of any patient were excluded from the study. With a handheld pH meter, urine pH is tested. Patients who match the criteria are given a genital wash and are then allowed to void midstream. The patient’s name and medical record number are written on the sterile container containing the urine before it is delivered to the microbiology lab. The pH of the urine is tested using a pH meter within the container. The procedure described is used to monitor the mean pH of each participant’s urine before administering intravesical MMC. Patients were categorized into two groups based on mean urinary pH. Patients with a mean urinary pH less than 5.5 were included in group A, which consisted of 26 patients, while patients with a mean urinary pH greater than 5.5 were included in group B, which consisted of 34 patients. Both groups of patients received intravesical MMC 40 mg diluted in 20 ml sterile water within 24 h following TURBT, and MMC instillation was given once weekly for 6 weeks. Both groups had similar demographic and clinicopathological profiles. Patients were evaluated by urine C/E and cystoscopy at 3, 6, and 12 months postoperatively. Recurrence-free survival rates (RFSs) were assessed for both groups of patients and compared with the Kaplan–Meir curve. Continuous variables like age were described as mean with SD. Kaplan–Meier curves were obtained using SPSS version 26. A *P* value less than 0.05 was considered statistically significant. COX hazard regression analysis was applied to gain an association between alkaline urine pH and the RFS.

This study followed the STROCESS 2021 checklist for cross-sectional studies^14^.

## Results

Sixty patients were enrolled. Group A had a urinary pH less than 5.5 and consisted of 26 patients. Thirty-four patients in group B had a target urinary pH greater than 5.5. The baseline demographic and clinicopathological profile are described in Table 1. The mean age of participants was 60.95±10.31 and 57.32±8.11 years in group A and group B, respectively. Male to female ratio was 2 : 1 vs. 17 : 13 in group A and group B, respectively. The baseline creatinine level was 0.8±0.10 mg/dl in group A and 0.9±0.7 mg/dl in group B group. Twenty-two patients had a smoking history in group B, while 19 patients in group A had a smoking history. Seventeen patients had stage Ta, and 13 had stage T1 on histopathology in group A, while 20 samples were Ta and 10 were T1 on histopathology in group B. Tumor size ranges from 31 to 61 mm in both groups (Table 1).

**Table 1:** Demographic and clinicopathological profile of patients

	Group A	Group B
Mean age (years)	60.95±10.31	57.32±8.11
Sex		
Male	20	17
Female	10	13
Baseline creatinine levels (mg/dl)	0.8±0.10	0.9±0.7
Tumor stage		
T1	13	10
Ta	17	20
Mean tumor size (31–61 mm)	47.15±8.584	42.15±3.125
Duration of follow-up in both groups (months)	15 (3–18)	15 (3–18)
Smoking history		
Yes	19	22
No	11	08
Mean pH	4.11±0.405	6.89±0.321

**FIGURE 1:**
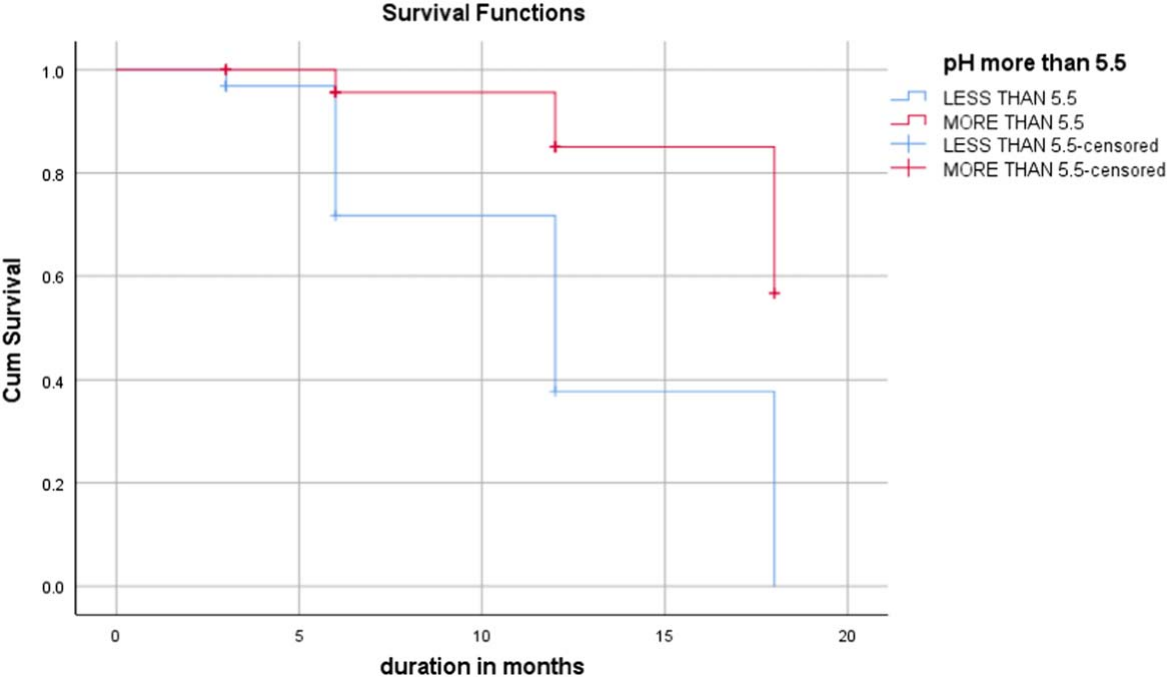
Comparison of Kaplan–Meier curves of intravesical mitomycin C with alkaline urine versus intravesical mitomycin C with acidic urine for delaying recurrence of bladder tissue after transurethral resection of bladder tumor.

The RFS rate was not associated with patient age (*P*=0.566), sex (*P*=0.293), and mean tumor size (*P*=0.417). However, cigarette smoking was associated with recurrence (*P*=0.030) (Table 2). The mean time to tumor recurrence in smokers versus nonsmokers was 13.27 and 16.029 months, respectively (*P*=0.030) (Table 3). In the patients in group B, the RFS rate at 3, 6, and 12 months was 95, 55, and 25%, respectively. In comparison, at corresponding intervals, RFS in group A was 60, 30, and 10%, respectively. There was a statistically significant difference between both two groups regarding RFS (Table 4). The mean time to tumor recurrence in group A and group B was calculated at 12.48 months, 95% CI (10.58–14.37) versus 16.84 months, 95% CI (14.99–18.69) by using COX hazard regression analysis. Univariate COX regression analysis predicted that alkaline urinary pH was an independent predictor of delaying recurrence in superficial bladder tumors (hazard coefficient *B*=−1.533, *P*=0.013, 95% CI: 1.43–1.71). A negative hazard coefficient is associated with increased survival rates. COX hazard regression analysis and Kaplan–Meier curves were employed to visualize the role of alkaline urinary pH (>5.5) in delaying tumor recurrence (Fig. 1).

**Table 2:** COX hazard regression analysis of different variables in delaying RFS rate of bladder tumor

Variables	*χ* ^2^	*P*
Age	24.178	0.566
Sex	1.107	0.293
Mean tumor size (mm)	23.764	0.417
Smoking	4.696	0.030

**Table 3:** Estimation of recurrence-free survival rate of bladder tumor in smokers

Smoking status	Estimate of recurrence-free survival rate (months)	*P*
No	16.029	0.030
Yes	13.274	

**Table 4:** Recurrence-free survival rates (RFS) in both groups at different time intervals

Recurrence-free survival rate (%)	Group A (Intravesical instillation of mitomycin C in patients with acidic urine) %	Group B (Intravesical instillation of mitomycin C in patients with alkaline urine) %	*P*
Within 3 months	60	95	0.021
Within 6 months	30	55	0.014
Within 12 months	10	25	0.052

## Discussion

MMC is an alkylating agent. MMC enters tumor cells on instillation and undergoes the process of chemical reduction to form an electrophilic intermediate that alkylates the cellular nucleophiles. MMC acts through various mechanisms, including alkylation, redox cycling, and cellular protein inhibition^15^. After resection of superficial bladder tumors, there is a 70% reported recurrence rate^16^. Over a time span of 3–24 months, MMC intravesical instillation decreases the rate of recurrence from 81 to 42.9%^17^. According to the current study, MMC instillation has greatly impeded the recurrence of 24-month follow-up^18^. According to Maeda *et al*.^7^ MMC’s stability and action are both pH dependent, and a urine pH of more than 5.5 indicates that a patient receiving MMC treatment has a good prognosis. Alkaline urine pH modifies the pharmacokinetics of MMC and stabilizes the drug. In contrast to MMC solution prepared in sterile water at higher concentrations, Beijnen and colleagues demonstrated that MMC solution designed in an alkaline solution is stable even at lower concentrations. Because MMC is more stable in an alkaline environment, this may be why there is an increased RFS rate in patients with alkaline urine^19^. Our study is synchronous with the findings of Maeda *et al*.^7^, who demonstrated that patients with nonmuscle-invasive BCa who completed intravesical MMC administration had a lesser probability of cancer recurrence when their urine pH was more than 5.5.

An increase in RFS following alkalinized MMC intravesical instillation is attributed to an increase in the stability of MMC at alkaline urine pH. At pH levels lower than 6, MMC rapidly loses its antitumor action. MMC is stable at higher pH (up to 10) and acceptable for intravesical administration^20^. There is a correlation between pH and MMC’s pharmacological activities. By restricting its degradation and absorption, urine alkalinization enhances MMC’s tumor exposure, and these findings have improved its anticancer activities. His results can be applied to our study, where urine alkalinization is associated with an increase in RFS^21^ After TURBT, it has been observed that 3% hypertonic saline can postpone the reemergence of superficial bladder tumors. Cell membranes are completely permeable to free water passage but are selectively permeable to various solutes. As a result, exposure of cells to hypertonic solutions forces water out of the intracellular space, causing cell destruction by osmotic dehydration^22^. Another study has claimed that saline infusion following TURBT for grade T1a tumor has RFS of 73 and 62% at 12 and 24 months^23^. Alkaline urine pH is associated with the formation of certain type of stones, like calcium phosphate, calcium carbonate, and magnesium phosphate stones. Neomycin, kanamycin, and streptomycin are effective in alkaline urine. Acidic pH modifies cellular dynamics, degrades the extracellular matrix, and enhances cellular motility^24^. Urinary pH below 5.5 is significantly associated with advanced urothelial tumors (T3–T4) relative to those above 5.5. Those patients with a urinary pH below 5.5 are more vulnerable to progressing to the advanced stage than those with a urine pH above 5.5. Han *et al*.^25^ has demonstrated that multifocal tumors, acidic urine pH, and advanced T-stage independently shorten recurrence-free survival. A study conducted by Wright *et al*.^26^ has reported the role of acidic urine pH in decreasing RFSs in patients with superficial bladder tumors, which is coherent with the finding concluded in the current study. The increased incidence of bladder tumors in acidic urine can be explained by the urogenous contact hypothesis, which demonstrates the increased binding of DNA to carcinogens like 4-aminobiphenyl in acidic urinary pH (27). Tumor cells become retained inside the bladder after TURBT. Later, these tumor cells adhere to each other and get covered with an extracellular matrix^27^. Hence, these neoplastic cells are the causative factors for the recurrence of NMIBC after TURBT. Some studies claim that acidic or alkaline urine has no association with the recurrence of bladder tumor following TURBT^28^. Urothelial carcinoma is most common in workers exposed to benzidine-based dyes. When these benzidine dyes are exposed to the inner cell lining of the bladder, there are 10 folds increased chances that these chemicals will form an adduct with bladder cell DNA at pH less than 6^29^. Patients with an active smoking history are more vulnerable to DNA-reactive aromatic amines in acidic urine at pH=6 or less than 6, thus promoting urothelial carcinoma^30^.

### Limitations

Given that this study was retrospective in nature and has a limited sample size, larger sample size studies with prospective study designs are advised in order to further confirm our findings. The retrospective element may add information bias, erroneousness, and selection bias. Selection bias is introduced in the current investigation by randomly assigning patients to either group based on their urine pH. We were only able to conduct a preliminary study with a small sample size due to a lack of resources (time and money). While some of the patients who visited us had ambigous urine pH values that reduced the quantity of the sample, the majority of them had invasive muscle illnesses.

Assessing the temporal relationship in retrospective research is frequently challenging. We have not undergone molecular markers, tumoroncogenes, tumor suppressor genes, and proliferative indexing due to the lack of these facilities in our healthcare facility.

## Conclusion

Patients receiving intravesical MMC with alkaline urine have improved RFSs compared to patients with an acidic urinary pH. By restricting its degradation and absorption, urine alkalinization enhances MMC’s tumor exposure, and these findings have improved its anticancer activities. A pH that is acidic impacts cellular dynamics, degrades the extracellular matrix, and accelerates cellular movement. Comparatively to urine pH levels over 5.5, advanced urothelial tumors (T3–T4) are strongly related to urine pH values below 5.5. The therapeutic effectiveness of MMC instillation may be enhanced through monitoring urinary pH during mitomycin C adjuvant therapy and altering pH for urine alkalization.

## Ethical approval

Ethical approval for this study (Ethical Committee N° ZMC/TERC/298/2022) was provided by the Institutional Review Board of Shaikh Zayed Hospital, Lahore, Pakistan, on 20 December 2022.

## Consent

Written informed consent was obtained from the patients for the publication and any accompanying images. A copy of the written consent is available for review by the Editor-in-Chief of this journal on request.

## Sources of funding

The authors received no financial support for the research and or authorship of this article.

## Author contribution

M.T.N.: concept and design of study. A.H.U. and S.A.: statistical analysis. H.R., A.N.R., M.M.F.E., and M.T.N.: drafting/writing. All authors approved the study.

## Conflicts of interests

The authors declared no potential conflicts of interest.

## Research registration unique identifying number (UIN)

1. Name of the registry: NA.
2. Unique identifying number or registration ID: NA.
3. Hyperlink to your specific registration (must be publicly accessible and will be checked): NA.

## Guarantor

Mohammed Mahmmoud Fadelallah Mohammed, Faculty of Medicine, University of Bakht Alruda, Ad Duwaym 11112, Sudan. Mobile: 00249964656914. E-mail: m.mahmmoud96@gmail.com.

## Availability of data and materials

The materials datasets used and/or analyzed during this study are available from the corresponding author upon reasonable request.

## Provenance and peer review

Not commissioned, externally peer-reviewed.
